# Elimination of Harmonic Force and Torque in Active Magnetic Bearing Systems with Repetitive Control and Notch Filters

**DOI:** 10.3390/s17040763

**Published:** 2017-04-04

**Authors:** Xiangbo Xu, Shao Chen, Jinhao Liu

**Affiliations:** School of Technology, Beijing Forestry University, No. 35 Tsinghua East Road, Haidian District, Beijing 100083, China; xuxiangbo@bjfu.edu.cn (X.X.); liujinhao_2016@163.com (J.L.)

**Keywords:** active magnetic bearing, harmonic force and torque, rotor imbalances, repetitive control, notch filter

## Abstract

Harmonic force and torque, which are caused by rotor imbalance and sensor runout, are the dominant disturbances in active magnetic bearing (AMB) systems. To eliminate the harmonic force and torque, a novel control method based on repetitive control and notch filters is proposed. Firstly, the dynamics of a four radial degrees of freedom AMB system is described, and the AMB model can be described in terms of the translational and rotational motions, respectively. Next, a closed-loop generalized notch filter is utilized to identify the synchronous displacement resulting from the rotor imbalance, and a feed-forward compensation of the synchronous force and torque related to the AMB displacement stiffness is formulated by using the identified synchronous displacement. Then, a plug-in repetitive controller is designed to track the synchronous feed-forward compensation adaptively and to suppress the harmonic vibrations due to the sensor runout. Finally, the proposed control method is verified by simulations and experiments. The control algorithm is insensitive to the parameter variations of the power amplifiers and can precisely suppress the harmonic force and torque. Its practicality stems from its low computational load.

## 1. Introduction

Control moment gyroscope (CMG) is one of the most important spacecraft attitude control actuators [1]. Extremely high stability of the spacecraft platform is indispensable for high-resolution Earth observation spacecraft equipped with many sensitive payloads. The CMG consists of a high-speed rotor mounted on a gimbal, which is fixed in the spacecraft platform [2]. The platform stability is severely affected by the undesirable harmonic force and torque of the high-speed rotor in the CMG [3]. Considering the support method of the high-speed rotor, CMGs can be divided into CMGs with mechanical bearings and CMGs with active magnetic bearings (AMBs) [4]. If mechanical bearings are employed, considerable harmonic force and torque will be directly transferred to the spacecraft platform [5]. In contrast, AMB, which provides several significant advantages of low friction, high speed, adjustable bearing damping, and especially active control ability, has been widely used to support the high-speed rotor of the CMG [6].

The frequencies of the harmonic force and torque in AMB are mainly composed of synchronous and multiple higher integer harmonics of the rotational speed [7]. Rotor imbalance, which results from the discrepancy between the geometric axis and the inertial axis of the rotor, is regarded as the main source of the synchronous force and torque source [8]. Suppression methods of the rotor imbalance fall into two main categories: suppression of displacement [9] and suppression of housing vibration [8]. The former one aims at forcing the rotor to rotate around its geometric axis [10], while the centrifugal force which quadratically rises with the rotational speed will induce severe housing vibrations. To solve this problem, the latter one makes the rotor rotate around the inertial axis. Several control methods have been reported to suppress the housing vibration by reducing synchronous current, such as generalized notch filters [8], Fourier coefficient computation [11], etc. In fact, the synchronous housing vibration is induced by the imbalance force and torque, which are composed of two parts related to the current stiffness and the displacement stiffness, respectively. Although the synchronous housing vibration related to the current stiffness can be well eliminated by using these synchronous current reduction strategies, a certain amount of housing vibration related to the displacement stiffness still remains [12]. For further suppression of the residual housing vibration, precise control current should be provided by designing additional controllers, such as adaptive autocentering control and feed-forward compensation [13,14]. Suppression of housing vibration is referred to as the elimination of synchronous force and torque strategy, which achieves a clean transmission of vibration force and torque. However, the suppression precision is severely affected by the parameter variations of power amplifiers in the AMB system [12]. Adaptive least mean square feed-forward [15], gain phase modifier [16] and double-loop compensation [17] have been proposed to achieve an adaptive compensation of the power amplifiers. However, it is very difficult to analyze the performance of these controllers owing to their complex algorithm, and intensive computational effort is required.

Sensor runout, which results from the non-uniform properties around the sensing surface of the rotor, also generates disturbances at multiple integer frequencies of the rotational speed [7]. Setiawan proposed a simultaneous identification and compensation method of the synchronous sensor runout and the rotor imbalance based on adaptive controller and bearing stiffness variation [18]. However, it is difficult to guarantee the stability of the adaptive algorithm and to analyze its performances. To identify or to suppress multi-frequency disturbances, compact wavelets [19], modified notch filter [20], response matching with FIR filter [21] and synchronous rotating frame transformation [22] can be employed, but the control’s computational complexity will sharply increase in direct proportion to the number of the harmonics considered. The computational cost may exceed the hardware capability if a four radial degrees of freedom (DOF) AMB system is studied. It is difficult even unavailable to run too much control algorithm in a very short sampling time for spatial microprocessors, because it has a low calculation capability [23]. To simplify the control algorithm, an iterative learning control was proposed to eliminate the unbalance effects by Bi [24], but only the translational motions in two radial DOF is considered. 

To precisely eliminate harmonic force and torque, the synchronous force and torque caused by the AMB displacement stiffness and rotor imbalance has to be compensated accurately and adaptively unaffected by the parameter variations of the power amplifiers, and the harmonic force and torque caused by the sensor runout have to be effectively suppressed simultaneously. For practical uses, the computation effort of the control algorithm has to be limited. The repetitive controller is widely employed owing to its superior tracking performance of periodic reference signal and low output total harmonic distortion [25]. Xu proposed a two-step suppression method of harmonic vibration [26]. Since the harmonic vibration is caused by the rotor imbalance and the sensor runout. To distinguish them, field balancing is firstly employed for online identification and offline compensation of the rotor imbalance. Once the rotor imbalance is reduced, the residual harmonic vibration is only the harmonic current, which is reduced by the sensor runout. Then a repetitive controller is designed to suppress the harmonic current. However, there are many problems in the field balancing in practice. Apparently, it is time-consuming because it needs to disassemble the AMB system and to take the rotor out for improving the mass distribution with discrete add-on weights. This will decrease the mechanical assembling accuracy. Moreover, a certain amount of the rotor imbalance always remains, and it changes during the operation [8].

In this work, a new method to eliminate harmonic force and torque in four radial-DOF AMBs has been proposed and studied. Field balancing or disassembly are no longer needed. Only the control algorithm is designed so that the rotor imbalance and sensor runout can be separated and suppressed on-line. Generalized notch filter combing with feed-forward compensator are formulated to identify the rotor imbalance and to reduce the synchronous force and torque. Meanwhile, a plugin repetitive controller, insensitive to the parameter variations of the power amplifiers, is designed to track the synchronous reference formulated by the identified rotor imbalance and to eliminate the harmonic force and torque caused by the sensor runout.

## 2. Harmonic Force and Torque of the 4-DOF AMB System

**Figure 1:**
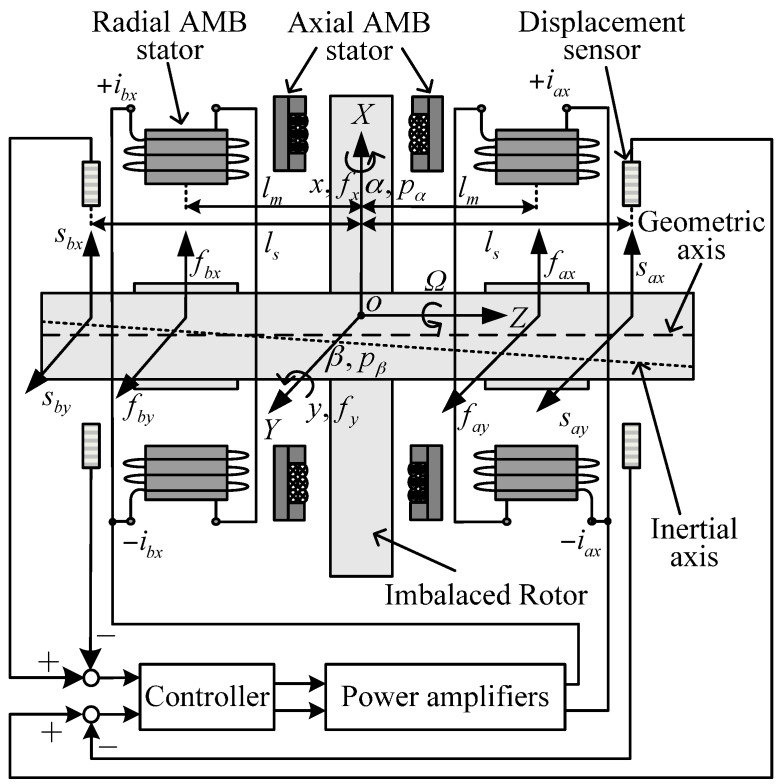
Diagram of the AMB system in the XZ plane.

Figure 1 shows the diagram of the AMB system in the XZ plane, which consists of an imbalanced rotor, four pairs of radial AMB stators and displacement sensors (only two of them are shown, and the others are oriented orthogonal to the paper), a controller and power amplifiers. 

According to the displacement of the rotor’s geometric axis, which can be measured by the displacement sensors, the controller drives the power amplifiers to generate control currents in the AMB stators. Then, magnetic force and torque are induced and controlled to levitate the rotor. As is known, every rotor has six DOF: three in terms of translational motions and three in terms of rotational motions. Among these freedoms in this AMB system, the axial rotational motion is driven by a motor with a speed of *Ω*, while the axial translational motion is controlled by the axial AMB. It is noted the harmonic force and torque have little couple with these two axial motions [7], so only four radial motions controlled by the radial AMBs are discussed in this work. To describe the AMB system, three coordinates are defined as:

(1) The generalized coordinate, where *o* the center of the AMB stators, *x* and *y* are the translational displacements in the *X* and *Y* directions, respectively, while $f_{x}$ and $f_{y}$ are the corresponding forces; *α* and *β* are the rotational displacements, while $p_{\alpha }$ and $p_{\beta }$ are the corresponding torques.
(2) The sensor coordinate, where $l_{s}$ is the distance from the center of a displacement sensor to the origin, $s_{ax}$, $s_{bx}$, $s_{ay}$ and $s_{by}$ are the measured displacements of the rotor’s geometric axis in four decentralized directions of *ax*, *bx*, *ay* and *by*, respectively.
(3) The stator coordinate, where $l_{m}$ is the distance from the center of a radial AMB stator to the origin, $i_{ax}$, $i_{bx}$, $i_{ay}$ and $i_{by}$ are the coil currents in the four decentralized directions (only $i_{ax}$ and $i_{bx}$ are visible in Figure 1), while $f_{ax}$, $f_{bx}$, $f_{ay}$ and $f_{by}$ are the corresponding control forces.

The displacements of the geometric and inertial axes are defined as $q_{G}={\left[x_{G}\;\beta_{G}\;y_{G}\;-\alpha_{G}\right]}^{T}$ and $q_{I}={\left[x_{I}\;\beta_{I}\;y_{I}\;-\alpha_{I}\right]}^{T}$ in the generalized coordinate. Since the static and dynamic imbalances are the eccentricity and inclination angle between $q_{G}$ and $q_{I}$, an imbalance vector Δq can be expressed by:

(1) $$\Delta q=q_{I}-q_{G}=\left[\begin{array}{c} \Delta x\\ \Delta \beta \\ \Delta y\\ -\Delta \alpha \end{array}\right]=\left[\begin{array}{c} \varepsilon \cos\left(\Omega t+\chi \right)\\ \sigma \sin\left(\Omega t+\delta \right)\\ \varepsilon \sin\left(\Omega t+\chi \right)\\ -\sigma \cos\left(\Omega t+\delta \right) \end{array}\right]$$

where ε (σ) and χ (δ) are the amplitude and the initial phase of the static (dynamic) imbalance.

Since senor runout is the displacement noise, its vector $q_{sr}$ is defined in the sensor coordinate. After compensating the synchronous component manually [18], residual $q_{sr}$ can be expressed as:

(2) $$q_{sr}=\left[\begin{array}{c} \sum_{i=2}^{n}s_{asi}\sin(i\Omega t+\alpha_{si})\\ \sum_{i=2}^{n}s_{bsi}\sin(i\Omega t+\beta_{si})\\ \sum_{i=2}^{n}s_{asi}\sin(i\Omega t+\alpha_{si}-\frac{i\pi }{2})\\ \sum_{i=2}^{n}s_{bsi}\sin(i\Omega t+\beta_{si}-\frac{i\pi }{2}) \end{array}\right]$$

where i is the harmonic number, $s_{asi}$ and $s_{bsi}$ are harmonic Fourier coefficients, $\alpha_{si}$ and $\beta_{si}$ are harmonic initial phases.

According to the gyro technique equations and Newton’s second law, the dynamics of the AMB system in the radial four DOF can be given by [26]:

(3) $$\left\{ \begin{array}{l} ms^{2}x_{I}\left(s\right)=2\left[k_{x}-2k_{i}k_{s}G_{w}\left(s\right)C_{t}\left(s\right)\right]\left[x_{I}\left(s\right)-\Delta x\left(s\right)\right]-2k_{i}G_{w}\left(s\right)C_{t}\left(s\right)x_{sr}\left(s\right)\\ ms^{2}y_{I}\left(s\right)=2\left[k_{x}-2k_{i}k_{s}G_{w}\left(s\right)C_{t}\left(s\right)\right]\left[y_{I}\left(s\right)-\Delta y\left(s\right)\right]-2k_{i}G_{w}\left(s\right)C_{t}\left(s\right)y_{sr}\left(s\right) \end{array}\right.$$

(4) $$\left\{ \begin{array}{c} J_{r}s^{2}\alpha_{I}\left(s\right)+J_{z}\Omega s\beta_{I}\left(s\right)=2\left[k_{x}{l_{m}}^{2}-k_{i}k_{s}l_{m}l_{s}G_{w}\left(s\right)C_{rs}\left(s\right)\right]\left[\alpha_{I}\left(s\right)-\Delta \alpha \left(s\right)\right]-2k_{i}l_{m}G_{w}\left(s\right)C_{rs}\left(s\right)\alpha_{sr}\\ +2k_{i}k_{s}l_{m}l_{s}G_{w}\left(s\right)C_{rc}\left(s\right)\left[\beta_{I}\left(s\right)-\Delta \beta \left(s\right)\right]+2k_{i}l_{m}G_{w}\left(s\right)C_{rc}\left(s\right)\beta_{sr}\\ J_{r}s^{2}\beta_{I}\left(s\right)-J_{z}\Omega s\alpha_{I}\left(s\right)=2\left[k_{x}{l_{m}}^{2}-k_{i}k_{s}l_{m}l_{s}G_{w}\left(s\right)C_{rs}\left(s\right)\right]\left[\beta_{I}\left(s\right)-\Delta \beta \left(s\right)\right]-2k_{i}l_{m}G_{w}\left(s\right)C_{rs}\left(s\right)\beta_{sr}\\ -2k_{i}k_{s}l_{m}l_{s}G_{w}\left(s\right)C_{rc}\left(s\right)\left[\alpha_{I}\left(s\right)-\Delta \alpha \left(s\right)\right]-2k_{i}l_{m}G_{w}\left(s\right)C_{rc}\left(s\right)\alpha_{sr} \end{array}\right.$$

where m is the mass of the rotor, $k_{x}$ is the AMB displacement stiffness, $k_{i}$ is the AMB current stiffness, $k_{s}$ is the coefficient of the displacement sensor, $G_{w}(s)$ is the transfer function of the simplified first-order low-pass filter model of the power amplifier, $J_{r}$ and $J_{z}$ are the transverse and polar moments of inertia of the rotor, respectively, $C_{t}(s)$ is the main controller of the translational system, $C_{rs}(s)$ and $C_{rc}(s)$ are the main controllers of the coupled rotational system, and:

$$G_{w}(s)=k_{w}\frac{\omega_{w}}{s+\omega_{w}},\{\begin{array}{l} C_{t}\left(s\right)=k_{P}+k_{I}\frac{1}{s}+k_{D}s\\ C_{rs}\left(s\right)=k_{P}+k_{I}\frac{1}{s}+k_{D}s+\frac{k_{rh}\Omega s}{s+\omega_{rh}}\cos\phi -\frac{k_{rl}\Omega \omega_{rl}}{s+\omega_{rl}}\cos\varphi \\ C_{rc}\left(s\right)=\frac{k_{rh}\Omega s}{s+\omega_{rh}}\sin\phi -\frac{k_{rl}\Omega \omega_{rl}}{s+\omega_{rl}}\sin\varphi \end{array},$$

$$\{\begin{array}{l} x_{sr}=\frac{1}{2}\sum_{i=2}^{n}\left[s_{asi}\sin(i\Omega t+\alpha_{s})+s_{bsi}\sin(i\Omega t+\beta_{s})\right]\\ y_{sr}=\frac{1}{2}\sum_{i=2}^{n}\left[s_{asi}\sin(i\Omega t+\alpha_{s}-\frac{i\pi }{2})+s_{bsi}\sin(i\Omega t+\beta_{s}-\frac{i\pi }{2})\right]\\ \alpha_{sr}=\frac{1}{2}\sum_{i=2}^{n}\left[s_{bsi}\sin(i\Omega t+\beta_{s}-\frac{i\pi }{2})-s_{asi}\sin(i\Omega t+\alpha_{s}-\frac{i\pi }{2})\right]\\ \beta_{sr}=\frac{1}{2}\sum_{i=2}^{n}\left[s_{asi}\sin(i\Omega t+\alpha_{s})-s_{bsi}\sin(i\Omega t+\beta_{s})\right] \end{array},\left\{ \begin{array}{l} \Delta x\left(s\right)=\varepsilon \frac{\cos\chi s-\Omega \sin\chi }{s^{2}+\Omega^{2}}\\ \Delta y\left(s\right)=\varepsilon \frac{\sin\chi s+\Omega \cos\chi }{s^{2}+\Omega^{2}}\\ \Delta \alpha \left(s\right)=\sigma \frac{\cos\delta s-\Omega \sin\delta }{s^{2}+\Omega^{2}}\\ \Delta \beta \left(s\right)=\sigma \frac{\sin\delta s+\Omega \cos\delta }{s^{2}+\Omega^{2}} \end{array}\right.$$

$k_{w}$ and $\omega_{w}$ are the gain and the cutoff frequency of the simplified low-pass power amplifier model, $k_{P}$, $k_{I}$ and $k_{D}$ are coefficients of the typical proportional-integral-derivative controller, $k_{rh}$ and $k_{rl}$ are gains of the cross feedback control to suppress the gyroscopic effect [27], $\omega_{rh}$ and $\omega_{rl}$ are the cutoff frequencies of the high-pass and low-pass filters, respectively, ϕ and φ are the cross phases. From Equations (3) and (4), the following conclusions can be drawn:

(1) The rotor imbalance generates synchronous force and torque related to both $k_{i}$ and $k_{x}$, whereas sensor runout generates multiple higher harmonic force and torque only related to $k_{i}$ due to its nature of the measured sensor noise. Therefore, they need different suppression methods. To suppress the synchronous force and torque, accurate control current should be generated so that the synchronous vibration related to $k_{i}$ counteracts that related to $k_{x}$ precisely. To suppress harmonic force and torque, only harmonic currents need be cleaned because they are only related to $k_{i}$.
(2) Since the employed $G_{w}$ is voltage-sourced, its parameter variations can highly decrease the precision of the synchronous control current. For a precise suppression of the synchronous force and torque, the synchronous control current has to be accurate, so that the parameter variations of $G_{w}$ can be well compensated.
(3) The translational motions and the rotational motions are uncoupled. Furthermore, the two translational motions are also uncoupled, whereas the two rotational motions are coupled because of the gyroscopic effects [27].

To simplify the 4-DOF AMB system, which can be transformed to two plural subsystems, we let:

(5) $$\{\begin{array}{c} r_{I}=x_{I}+jy_{I}\\ \begin{array}{l} \Delta r=\Delta x+j\Delta y\\ r_{sr}=x_{sr}+jy_{sr} \end{array}\\ {ο}_{I}=\alpha_{I}+j\beta_{I}\\ \begin{array}{l} \Delta ο=\Delta \alpha +j\Delta \beta \\ {ο}_{sr}=\alpha_{sr}+j\beta_{sr} \end{array} \end{array}$$

where j is the complex unit.

Then Equations (3) and (4) can be expressed as:

(6) $$ms^{2}r_{I}\left(s\right)=-2k_{i}G_{w}\left(s\right)C_{t}\left(s\right)r_{sr}\left(s\right)+2\left[k_{x}-2k_{i}k_{s}G_{w}\left(s\right)C_{t}\left(s\right)\right]\left[r_{I}\left(s\right)-\Delta r\left(s\right)\right]$$

(7) $$J_{r}s^{2}o_{I}\left(s\right)-jJ_{z}\Omega so_{I}\left(s\right)=-2k_{i}l_{m}G_{w}\left(s\right)C_{sc}\left(s\right)o_{sr}+2\left[k_{x}{l_{m}}^{2}-k_{i}k_{s}l_{m}l_{s}G_{w}\left(s\right)C_{sc}\left(s\right)\right]\left[o_{I}\left(s\right)-\Delta o\left(s\right)\right]$$

where:

$$\{\begin{array}{l} \Delta r\left(s\right)=\frac{\varepsilon e^{j\chi }}{s-j\Omega }\\ \Delta ο\left(s\right)=\frac{\sigma e^{j\delta }}{s-j\Omega } \end{array}$$

$$C_{sc}\left(s\right)=C_{rs}\left(s\right)+jC_{rc}\left(s\right)=C_{t}\left(s\right)+\left(k_{rh}\frac{s}{s+\omega_{rh}}e^{j\phi }-k_{rl}\frac{\omega_{rl}}{s+\omega_{rl}}e^{j\varphi }\right)\Omega$$

If a complete suppression of harmonic force and torque can be achieved, the rotor will rotate around its inertial axis, which means $r_{I}$ and $o_{I}$ are zero. Then, we can see from Equations (6) and (7) that the left sides of both equations are equal to zero. The right sides of both equations are composed of two parts: the sensor runout ($r_{sr}$ and $o_{sr}$) part only related to $k_{i}$ and the rotor imbalance (Δr and Δo) part related to both $k_{i}$ and $k_{x}$. Therefore, to suppress the synchronous force and torque, synchronous control current should be generated so that two synchronous vibrations respectively related to $k_{i}$ and $k_{x}$ counteract. Moreover, synchronous control current should be precise unaffected by parameter variations of $G_{w}(s)$. To suppress the multiple higher harmonic force and torque, only a reduction of the harmonic currents is needed.

## 3. Suppression of the Harmonic Force and Torque

**Figure 2:**
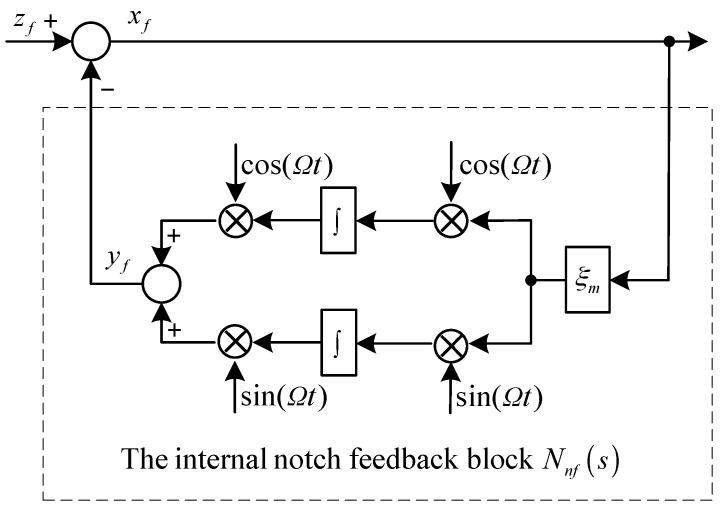
Structure of the closed-loop generalized notch filter.

The structure of the closed-loop generalized notch filter with an internal notch feedback block [8] is shown in Figure 2, where $N_{nf}\left(s\right)$ is the transfer function of the internal notch feedback, $z_{f}$ is the input signal with a synchronous component to be separated, $y_{f}$ is the output signal, $x_{f}$ is error signal, $\xi_{m}$ is the damping coefficient. The dynamic equation of the internal feedback block can be given by:

(8) $$y_{f}=\xi_{m}\left[\begin{array}{cc} \sin\left(\Omega t\right) & \cos\left(\Omega t\right) \end{array}\right]\int \left[\begin{array}{c} x_{f}\sin\left(\Omega t\right)\\ x_{f}\cos\left(\Omega t\right) \end{array}\right]dt$$

From Equation (8), the following equations can be easily verified:

(9) $$\{\begin{array}{l} y_{f}=\xi_{m}\left[\sin(\Omega t)\int x_{f}\sin(\Omega t)dt+\cos(\Omega t)\int x_{f}\cos(\Omega t)dt\right]\\ {\ddot{y}}_{f}+\Omega^{2}y_{f}=\xi_{m}{\dot{x}}_{f} \end{array}$$

Then we can obtain the transfer function as:

(10) $$N_{nf}\left(s\right)=\frac{y_{f}\left(s\right)}{x_{f}\left(s\right)}=\xi_{m}\frac{s}{s^{2}+\Omega^{2}}$$

From Equation (10), it’s easy to verify that the magnitude at the notch frequency of *Ω* is infinite. The transfer function of the closed-loop generalized notch filter can be given:

(11) $$G_{nf}\left(s\right)=\frac{x_{f}\left(s\right)}{z_{f}\left(s\right)}={\left(1+N_{nf}\left(s\right)\right)}^{-1}=\frac{s^{2}+\Omega^{2}}{s^{2}+\xi_{m}s+\Omega^{2}}$$

It is clear that $G_{nf}\left(s\right)$ will vanish if s=jΩ and $\xi_{m}\neq 0$, and this confirms its notch filter characteristics.

Upon the convergence of the closed-loop generalized notch filer, $y_{f}$ will be the separated synchronous component of $z_{f}$. Therefore, $y_{f}$ can be utilized to design the feedforward compensation, which will generate the synchronous control current to counteract the synchronous force and torque related to $k_{x}$.

**Figure 3:**
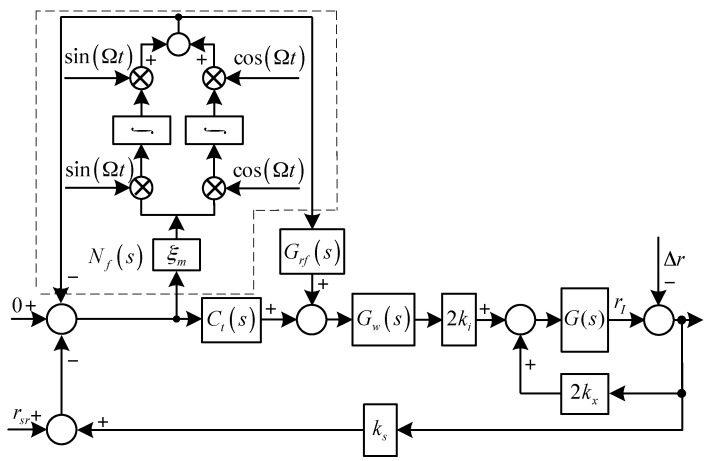
Diagram of the translational system with synchronous force elimination.

**Figure 4:**
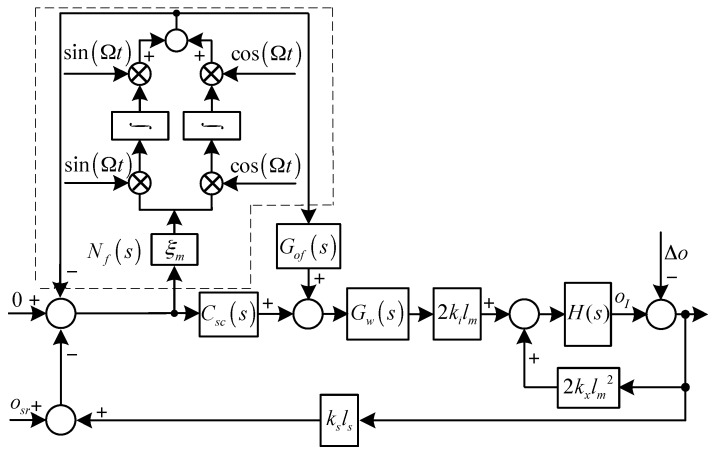
Diagram of the rotational system with synchronous torque elimination.

The diagrams of the translational system and the rotational system with synchronous force and torque elimination are shown in Figure 3 and Figure 4, respectively, where $G_{rf}\left(s\right)$ and $G_{of}\left(s\right)$ are the feed-forward controllers for the translational an rotational systems, respectively, and:

$$G\left(s\right)=\frac{1}{ms^{2}}$$

$$H\left(s\right)=\frac{1}{J_{r}s^{2}-jJ_{z}\Omega s}$$

As shown in Figure 3 and Figure 4, to eliminate the synchronous force and torque, we have:

(12) $$\{\begin{array}{c} \underset{s=j\Omega }{\lim}\frac{r_{I}(s)}{G\left(s\right)}(s-j\Omega )=\underset{s=j\Omega }{\lim}-\frac{C_{r0}(s)\Delta r\left(s\right)}{1-G(s)C_{r0}(s)}(s-j\Omega )=0\\ \underset{s=j\Omega }{\lim}\frac{o_{I}(s)}{H\left(s\right)}(s-j\Omega )=\underset{s=j\Omega }{\lim}-\frac{C_{o0}(s)\Delta o\left(s\right)}{1-H(s)C_{o0}(s)}(s-j\Omega )=0 \end{array}$$

where:

$$C_{r0}(s)=2k_{x}-2k_{i}k_{s}G_{w}(s)\left[G_{nf}(s)C_{t}(s)+\left(1-G_{nf}(s)\right)G_{rf}(s)\right]$$

$$C_{o0}(s)=2k_{x}l_{m}^{2}-2k_{i}k_{s}l_{m}l_{s}G_{w}(s)\left[G_{nf}(s)C_{sc}(s)+\left(1-G_{nf}(s)\right)C_{of}(s)\right]$$

Solving Equation (12) yields:

(13) $$\{\begin{array}{c} {C_{rf}(s)|}_{s=j\Omega }=\frac{k_{x}}{k_{i}k_{s}}G_{w}{\left(j\Omega \right)}^{-1}\\ {C_{of}(s)|}_{s=j\Omega }=\frac{k_{x}l_{m}}{k_{i}k_{s}l_{s}}G_{w}{\left(j\Omega \right)}^{-1} \end{array}$$

In practice, it is difficult to design $G_{w}{\left(j\Omega \right)}^{-1}$ and to keep it accurate all the time, because parameter variations will inevitably occur during operation (e.g., thermal effects).

For a good elimination of the harmonic force and torque, the synchronous feed-forward control current has to stay precise unaffected by the parameter variations of $G_{w}$, while an accurate reduction of the higher harmonic currents is achieved. Repetitive control is employed owing to its superior tracking performance of the periodic reference signal and attenuation performance of the harmonic disturbance signals. 

**Figure 5:**
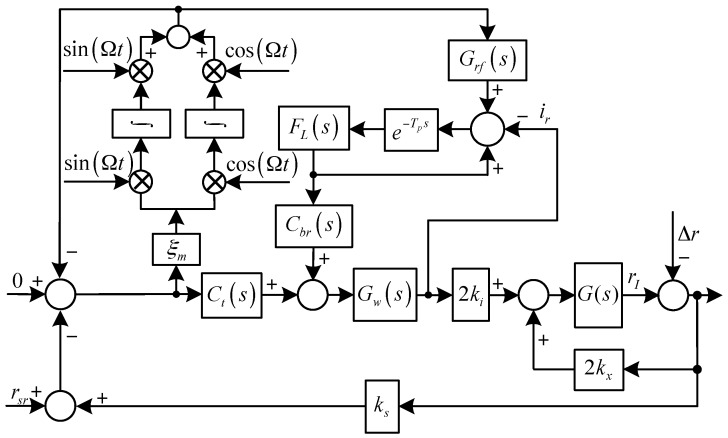
Diagram of the translational system with harmonic force elimination.

**Figure 6:**
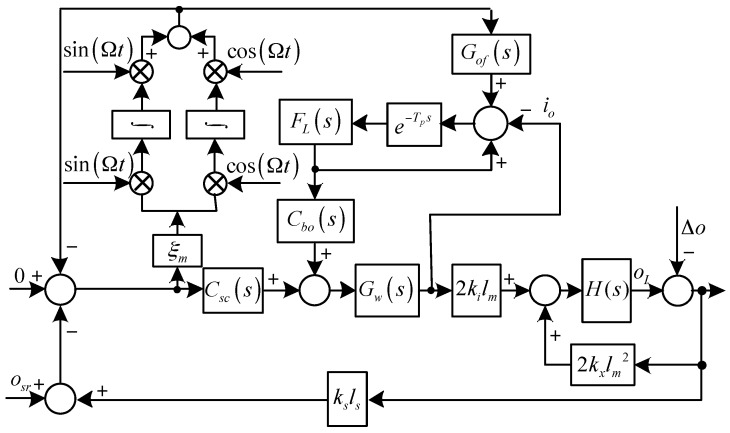
Diagram of the rotational system with harmonic torque elimination.

Figure 5 and Figure 6 show the diagrams of the translational and rotational systems with harmonic force and torque elimination, where $e^{-T_{p}s}$ is a delay element, and *T_p_* is the delay. It is expected from the internal model principle that he harmonic disturbance signals can be well suppressed if *T_p_* is equal to the period of the rotor speed. $F_{L}\left(s\right)$ is a low-pass filter to improve the system stability, $C_{br}\left(s\right)$ and $C_{bo}\left(s\right)$ are lead elements to improve the system bandwidth, $i_{r}$ is the translational current and $i_{r}\;=\;i_{x}+\;ji_{y}$, $i_{o}$ is the rotational current and $i_{o}\;=\;i_{\alpha }+\;ji_{\beta }$:

$$F_{L}\left(s\right)=\frac{\omega_{L}}{s+\omega_{L}}$$

$$C_{br}\left(s\right)=k_{cr}\frac{s+\omega_{w}}{k_{\omega }s+\omega_{w}}$$

$$C_{bo}\left(s\right)=k_{co}\frac{s+\omega_{w}}{k_{\omega }s+\omega_{w}}$$

$\omega_{L}$ is the cutoff frequency of $F_{L}\left(s\right)$, $k_{cr}$ and $k_{co}$ are positive parameters to be chosen, $k_{\omega }$ is a positive parameter to compensate the phase lag due to $G_{w}$.

$i_{r}$ and $i_{o}$ are adopted as the feedback signals, so the equivalent harmonic disturbance currents caused by sensor runout can be well reduced. The feed-forward compensations, which are the synchronous control currents to compensate the synchronous vibration force and torque related to $k_{x}$, are used as the reference signals of the repetitive controllers, so that they can be precisely tracked unaffected by the parameter variations of $G_{w}$.

From Equation (13) and Figure 5 and Figure 6, we have:

(14) $$\{\begin{array}{c} C_{rf}(s)=\frac{k_{x}}{k_{i}k_{s}}\\ C_{of}(s)=\frac{k_{x}l_{m}}{k_{i}k_{s}l_{s}} \end{array}$$

The sensitive suppression functions of the repetitive controllers can be calculated as:

(15) $$\{\begin{array}{c} M_{r}\left(s\right)=\frac{1-F_{L}\left(s\right)e^{-T_{p}s}}{1-\left(1-\frac{C_{br}\left(s\right)G_{w}\left(s\right)}{1+C_{fr}\left(s\right)G_{w}\left(s\right)}\right)F_{L}\left(s\right)e^{-T_{p}s}}\\ M_{o}\left(s\right)=\frac{1-F_{L}\left(s\right)e^{-T_{p}s}}{1-\left(1-\frac{C_{bo}\left(s\right)G_{w}\left(s\right)}{1+C_{fo}\left(s\right)G_{w}\left(s\right)}\right)F_{L}\left(s\right)e^{-T_{p}s}} \end{array}$$

where:

$$C_{fr}\left(s\right)=2k_{i}k_{s}\frac{C_{t}\left(s\right)G\left(s\right)}{1-2k_{x}G\left(s\right)}$$

$$C_{fo}\left(s\right)=2k_{i}k_{s}l_{m}l_{s}\frac{C_{sc}\left(s\right)H\left(s\right)}{1-2k_{x}{l_{m}}^{2}H\left(s\right)}$$

To eliminate the harmonic force and torque, the sensitive suppression functions at the harmonic frequencies should be zero

(16) $$\underset{s=j2m\pi /T_{p}}{\lim}\left|M_{r}\left(s\right)\right|=\underset{s=j2m\pi /T_{p}}{\lim}\left|M_{o}\left(s\right)\right|=0$$

where $m=1,\;2,\;\cdots \;,\;m_{\max}$ with $m_{\max}$ the largest number of the harmonics to be suppressed.

To suppress the largest harmonic effectively, $\omega_{w}/k_{\omega }\geq m_{\max}\Omega$, then we have:

(17) $$k_{\omega }\leq \omega_{w}/\left(m_{\max}\Omega \right)$$

Solving Equation (16) yields:

(18) $$\underset{s=j2m\pi /T_{p}}{\lim}1-F_{L}\left(s\right)=0$$

Equation (17) can be divided into two conditions in terms of amplitude and phase, respectively.

(19) $$\left\{ \begin{array}{l} \underset{s=j2m\pi /T_{p}}{\lim}\left|F_{L}\left(s\right)\right|=1\\ \underset{s=j2m\pi /T_{p}}{\lim}∠F_{L}\left(s\right)e^{-T_{p}s}=0 \end{array}\right.$$

To fulfill the amplitude and phase requirements of Equation (19), $\omega_{L}$ and $T_{p}$ can be determined by:

(20) $$\{\begin{array}{c} \omega_{L}>2m_{m}\pi /T_{p}\\ T_{p}=\frac{2\pi }{\Omega }\left[1-\frac{1}{2\pi }\tan^{-1}\left(\frac{\Omega }{\omega_{L}}\right)\right] \end{array}$$

Since the control algorithm is implemented digitally, *T_p_* should be an integer multiple of the sampling period. To fulfill this condition, the value of $\omega_{L}$ can be tuned slightly within its value range.

The regeneration spectrum method can be utilized to analyze the system stability and to choose the values of $k_{cr}$ and $k_{co}$. However, it is time consuming. In fact, if $k_{cr}=k_{co}=0$, the repetitive controllers will be shut down. With the increase of the values of $k_{cr}$ and $k_{co}$, convergence speeds of $i_{x}$ and $i_{o}$ become higher, whereas the stability margins become smaller. Therefore, the actual values of $k_{cr}$ and $k_{co}$ have to be determined according to the performance in simulations and experiments.

## 4. Simulations and Experiments

**Figure 7:**
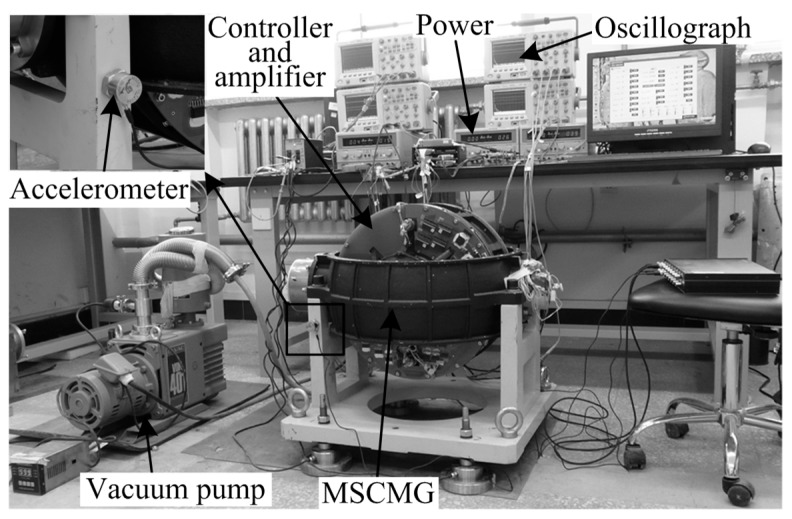
The picture of the experiment setup.

To verify the proposed control approach, simulations and experiments by using a magnetically suspended CMG (MSCMG), whose rotor is levitated by the AMB, have been performed. Figure 7 shows the picture of the experimental setup, which is composed of a vacuum pump, accelerometer, controller and amplifier, power, oscillograph, and MSCMG. The MSCMG consists of a gyro housing and a gimbal. The gimbal is supported by a bracket, where an accelerometer is employed to measure the harmonic vibration acceleration transmitted through the bracket to the spacecraft. A high-speed AMB system is inside the gyro room, while the vacuum pump is employed to create a nearly vacuous environment to reduce the wind resistance (the air pressure is about 2 Pa). The proposed control algorithm is implemented in a digital signal processor and field programmable gate array based controller with a sampling and control period of 148.6 μs. Eight eddy-current sensors are employed to measure $q_{G}$, while one Hall sensors is utilized to measure *Ω*. The oscillographs are employed to show, analyze, and store the values of the measured displacement, current and acceleration signals.

The parameters of the AMB system are presented in Table 1, where the values of the parameter are measured or estimated through actual experiments. It is noted that since the nominal speed of MSCMG is 200 Hz, while the first, third and fifth harmonics are dominant in practical experiments, the harmonic frequencies of 200, 600 and 1000 Hz are considered.

Only results related to *f_x_* and *p_α_* are shown here because they have the same amplitudes as *f_y_* and *p_β_*, respectively, except for steady phase lead angles of π/2, if the rotor rotates anticlockwise. Since very big differences exist in the amplitudes of different harmonics, their fast Fourier transformation (FFT) is carried out so that the harmonics of *f_x_* and *p_α_* can be easily recognized. Furthermore, to simulate the influence of the actual current noises on the performance of the proposed control method, a random noise with mean and variance values of 0 and 1 × 10^−4^ is added to the outputs of the power amplifiers.

**Figure 8:**
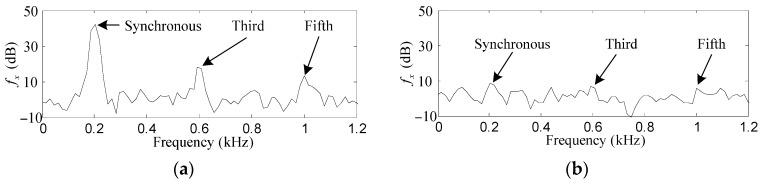
Simulation results of *f_x_*. (**a**) before harmonic force elimination; (**b**) after harmonic force elimination.

**Figure 9:**
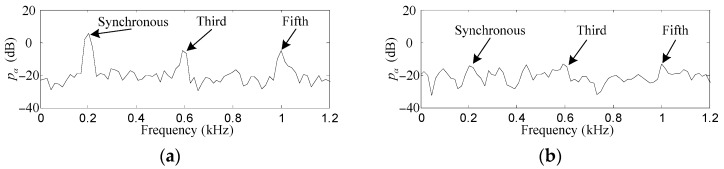
Simulation results of *p_α_*. (**a**) before harmonic torque elimination; (**b**) after harmonic torque elimination.

As shown in Figure 8a and Figure 9a, the synchronous, third and fifth harmonics of *f_x_* and *p_α_* without the proposed control method are very obvious among all the frequencies. The original synchronous, third and fifth harmonics of *f_x_* are 41.7, 17.8 and 13.1 dB, respectively, while those of *p_α_* are 5.4, −5.8 and −5.6 dB, respectively.

After the proposed control method is enabled, as can be seen in Figure 8b and Figure 9b, the synchronous, third and fifth harmonics of *f_x_* are suppressed to 8.6, 6.7 and 5.4 dB, respectively, while those of *p_α_* are suppressed to −14.2, −13.1 and −13.5 dB, respectively. The harmonics of *f_x_* and *p_α_* are suppressed by such considerable degrees that it is a little difficult to identify them among the random noise.

The magnetic force in the stator coordinate can be linearized as a function of coil current and the geometric axis displacement at the equilibrium point as [14]:

(21) $$f_{cn}=k_{i}i_{cn}+k_{x}x_{cn}$$

where *cn* is the channel number and cn=ax,bx,ay,by. $x_{cn}$ is the geometric axis displacement in the stator coordinate, and it can be obtained from the measured displacements in the sensor coordinate through a coordinate transformation.

(22) $$\left[\begin{array}{c} x_{ax}\\ x_{bx}\\ x_{ay}\\ x_{by} \end{array}\right]=\frac{1}{2k_{s}l_{s}}\left[\begin{array}{cccc} l_{s}+l_{m} & l_{s}-l_{m} & 0 & 0\\ l_{s}-l_{m} & l_{s}+l_{m} & 0 & 0\\ 0 & 0 & l_{s}+l_{m} & l_{s}-l_{m}\\ 0 & 0 & l_{s}-l_{m} & l_{s}+l_{m} \end{array}\right]\left[\begin{array}{c} s_{ax}\\ s_{bx}\\ s_{ay}\\ s_{by} \end{array}\right]$$

Then the force and torque in the generalized coordinate can be derived through another coordinate transformation as follows:

(23) $$\left[\begin{array}{c} f_{x}\\ p_{\beta }\\ f_{y}\\ -p_{\alpha } \end{array}\right]=\left[\begin{array}{cccc} 1 & 1 & 0 & 0\\ l_{m} & -l_{m} & 0 & 0\\ 0 & 0 & 1 & 1\\ 0 & 0 & l_{m} & -l_{m} \end{array}\right]\left[\begin{array}{c} f_{ax}\\ f_{bx}\\ f_{ay}\\ f_{by} \end{array}\right]$$

Finally, the values of *f_x_* and *p_α_* can be well acquired in experiments, as $i_{cn}$ and $s_{cn}$ are precisely measured by using Hall current sensors and eddy-current sensors.

**Figure 10:**
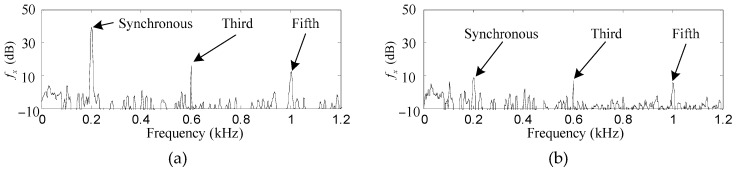
Experiment results of *f_x_*. (**a**) before harmonic force elimination; (**b**) after harmonic force elimination.

**Figure 11:**
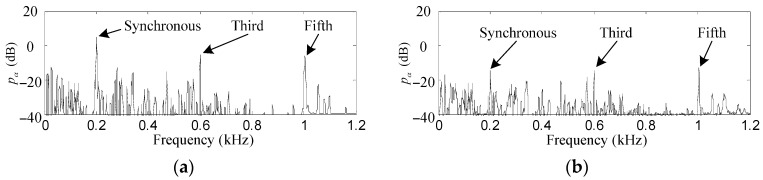
Experiment results of *p_α_*. (**a**) before harmonic torque elimination; (**b**) after harmonic torque elimination.

As shown in Figure 10a and Figure 11a, the first, third and fifth harmonics are distinct among the FFT of *f_x_* and *p_α_* without the proposed control method, their original values were about 39.8, 16.2, 12.4 dB and 5.2, −5.4, −5.7 dB, respectively. After the proposed control method is activated, as can be seen in Figure 10b and Figure 11b, those harmonic values of *f_x_* and *p_α_* are suppressed by 31, 9.1, 6.7 dB and 18.9, 8.9, 7.5 dB, respectively. Furthermore, good matching of the suppression degrees of the experiment results and the simulation results can be observed.

**Figure 12:**
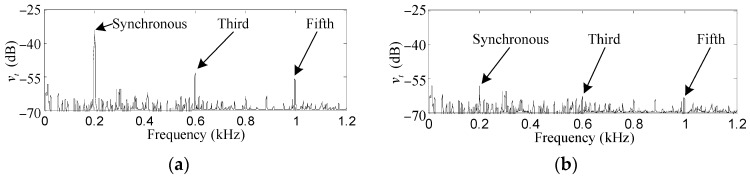
Experiment results of *V_t_*. (**a**) before harmonic force and torque elimination; (**b**) after harmonic force and torque elimination.

To give an independent verification of the practicality of the proposed control method, the measured acceleration *v_t_* by the accelerometer is used to demonstrate the vibration transmission from the AMB system to the bracket of the MSCMG. Comparing Figure 12a,b, the values of first, third and fifth harmonic vibrations are reduced from −33.8, −53.4 and −56.1 dB to −58.6, −62.7 and −63.1 dB, respectively. The residual harmonics have similar sizes to the visible noises (mainly caused by the gyroscopic effects and the structural resonance of the MSCMG test rig), which means the transmission of the harmonic vibrations is significantly attenuated.

Little change happens to the elimination precision of the harmonic vibrations during a ten-hour operation. This indicates that the repetitive controller is insensitive to the parameter variations caused by the temperature change. Compared with the adaptive synchronous compensation, which is nonlinear and bring difficulty in analyzing the closed-loop system stability [28], the repetitive controller is simpler. Furthermore, no additional computation is needed in this work, whereas the adaptive synchronous compensation is composed of many complex calculations, such as arc tangent and modulus operations. Compared with the harmonic vibration suppression in [26], only improvement of the control algorithm is needed. Since field balancing is no longer required, this will save a lot of time in practice. Furthermore, it is helpful for a good mechanical assembly accuracy without disassembly.

## 5. Conclusions

In this work, harmonic force and torque elimination in the 4-DOF AMB system with rotor imbalance and sensor runout is studied. A novel control method consisting of generalized notch filter, feed-forward compensation and repetitive control is proposed, and its effectiveness has been demonstrated by simulations and experiments. The first, third and fifth harmonic force and torque are well suppressed, and no visible mutual couplings among the harmonics or the other frequencies exist. The proposed method is very suitable for moment exchange devices with AMB systems in the high-resolution Earth observation spacecraft, and it can be extended to many industrial AMB applications, where suppression of the harmonic house vibration is needed. However, the suppression degree decreases with the increase of the frequency, this is mainly because the error between $F_{L}\left(s\right)e^{-Ts}$ and 1 becomes larger. The power spectrum, which acquires better estimation from noisy signals, is more appropriate for the data processing than the FFT. Improvement of the repetitive controller and data processing with the power spectrum will be topics for our further research work.

## Figures and Tables

**Table 1:** Parameters of the AMB system with the proposed control approach.

Parameters	Value	Parameters	Value
m	57 kg	$\;\xi_{m}$	2 × 10^6^
$J_{r}$	0.62 $\mathrm{kg}\cdot m^{2}$	Ω	200 Hz
$J_{z}$	0.82 $\mathrm{kg}\cdot m^{2}$	$\omega_{L}$	10^4^ rad/s
$l_{m}$	0.113 m	$T_{p}$	0.0049 s
$l_{s}$	0.178 m	$k_{cx}$	960
$k_{i}$	450 N/A	$k_{co}$	1100
$k_{x}$	2.5 × 10^6^ N/m	ε	5 × 10^−6^ m
$k_{s}$	1.5 × 10^7^ V/m	χ	π/3 rad
$\omega_{w}$	1683 rad/s	σ	2.8 × 10^−5^ rad
$k_{w}$	1.23 × 10^−4^ A/V	δ	−π/3 rad
$k_{P}$	5	$s_{as3}$	4
$k_{I}$	40	$\alpha_{s3}$	4π/3 rad
$k_{D}$	0.01	$s_{as5}$	1
$k_{rh}$	0.01	$\alpha_{s5}$	9π/5 rad
$k_{rl}$	0.001	$s_{bs3}$	5
$\omega_{rh}$	1256.6 rad/s	$\beta_{s3}$	11π/6 rad
$\omega_{rl}$	314.2 rad/s	$s_{bs5}$	2
ϕ	2.5 rad	$\beta_{s5}$	π/5 rad
φ	0.9 rad		
